# *Pseudomonas fluorescens* G3 Enhances the Salt Stress Tolerance of Maize and Modulates Soil Microbial Community Composition in the Rhizosphere

**DOI:** 10.3390/plants15091281

**Published:** 2026-04-22

**Authors:** Yue Lou, Chenying Wu, Xu Wang, Meiling Shi, Zhaoyu Li, Xu Su, Wenshuo Ye, Caiping Dai, Yongqiang Tian, Yang Liu

**Affiliations:** 1 School of Biological and Pharmaceutical Engineering, Lanzhou Jiaotong University, Lanzhou 730070, China; ly19861828217@126.com (Y.L.); wcy449315547@163.com (C.W.); xuxuxuuuu@126.com (X.W.); shiml@mail.lzjtu.cn (M.S.); lizy@mail.lzjtu.cn (Z.L.); 15353930905@163.com (W.Y.); 15117046096@163.com (C.D.); 2 Key Laboratory of Biodiversity Formation Mechanism and Comprehensive Utilization of the Qinghai-Tibet Plateau in Qinghai Province, Qinghai Normal University, Xining 810008, China; xusu8527972@126.com

**Keywords:** maize, salt stress, *Pseudomonas fluorescens*, rhizosphere microbial community, bioremediation

## Abstract

Soil salinization impacts over one billion hectares, threatening global food security. Here, a salt-tolerant bacterial strain, *Pseudomonas fluorescens* G3, was isolated from the rhizosphere of maize (Jinongyu-719) growing in saline–alkali soils in Gansu Province, China. This strain demonstrated the ability to secrete indole-3-acetic acid (IAA), 1-aminocyclopropane-1-carboxylic acid (ACC) deaminase, and extracellular polysaccharides. It also exhibited notable phosphate-solubilizing activity and robust siderophore production capabilities. Under salt stress conditions (200 mM NaCl), the *P. fluorescens* G3 strain significantly improved maize’s growth parameters, namely its plant height, root length, and dry weight. Further, it enhanced antioxidant enzyme activity while reducing the accumulation of malondialdehyde (MDA), mitigating stress-induced oxidative damage. In *P. fluorescens* G3-inoculated plants, leaf and root Na^+^ contents decreased by 34.90% and 33.91%, while their K^+^ contents increased by 40.20% and 33.47%, respectively. Inoculation with *P. fluorescens* G3 enhanced taxonomic richness (ACE, Chao1) and evenness (Shannon, Simpson) in the rhizosphere bacterial community, leading to a significantly greater relative abundance of several bacterial genera: *Pseudomonas*, *Methylophaga*, *Enhygromyxa*, *Desulfuromonas*, and *Devosia*. These shifts in the microbial community composition suggest a potential restructuring of functional profiles, possibly enhancing processes beneficial to plant salt tolerance, such as ion homeostasis and stress mitigation: the biosynthesis of cofactors and secondary metabolites; bacterial secretion and two-component systems; porphyrin metabolism; flagellar assembly; biofilm formation; and bacterial chemotaxis. Redundancy analysis revealed positive correlations between microbial composition at both the phylum and genus levels and the activity of stress resistance enzymes after treatment with *Pseudomonas fluorescens*. This study provides important theoretical foundations and microbial resources for utilizing microbial community regulation in saline–alkali soil bioremediation.

## 1. Introduction

Soil salinization now affects over one billion hectares of land globally, severely limiting crop productivity and threatening food security. According to the Food and Agriculture Organization (FAO) [1], soil salinization has caused productivity declines in approximately 20% of the world’s irrigated agricultural land (64.8 million hectares), with annual losses of maize (*Zea mays*) that could feed 135 million people [1]. Excess salt harms plants via Na^+^-induced osmotic stress, ion toxicity (Na^+^/K^+^ imbalance), and ROS bursts resulting in oxidative damage, membrane dysfunction, and photosynthetic inhibition, all of which could lead to less biomass and death in the absence of salt-tolerance mechanisms [2,3,4,5]. Maize, as a staple crop for food, feed, and industrial raw materials, plays a crucial role in maintaining the global food supply chain through its high and stable yields [6]. However, maize is a salt-sensitive crop whose germination, seedling establishment, growth, and reproduction are markedly reduced by high salinity, resulting in considerable yield losses [7,8]. According to FAO’s assessment [9], such yield reductions in maize—a core cereal whose global annual production exceeds 1.2 billion tons—can reach 40–60% due to its salt-sensitive traits.

To remediate saline–alkali soil, various treatment approaches have been developed that rely on physical, chemical, or biological means [9,10]. Physical methods—such as drainage and salt leaching, deep tillage—enhance soil permeability to flush out the salts, rapidly mitigating topsoil salinity in the short term [11]. Yet these approaches only work within well-established irrigation and drainage systems, consume vast amounts of water, and are only suitable for flat terrain; their prolonged use may raise groundwater levels, triggering secondary salinization [12]. Chemical remediation focuses on applying soil amendments, e.g., lime to neutralize exchangeable aluminum/hydrogen ions in acidic–saline–alkali soils, or gypsum to improve the physical structure of alkaline soils. Such interventions can rapidly adjust the soil’s pH and ionic composition [13]. However, the high cost of amendments and excessive applications may lead to soil compaction, heavy metal accumulation, and groundwater contamination, posing major secondary environmental risks [13]. Biological remediation utilizes salt-tolerant plants (e.g., Suaeda salsa, Tamarix chinensis) combined with organic materials (farmyard manure, biochar) to enhance soil organic matter, offering environmental compatibility advantages. Nevertheless, this approach requires prolonged remediation periods, and the slow biomass accumulation of halophytes makes it difficult to meet food crop production demands [14]. In summary, conventional strategies typically face recognized limitations, namely, prolonged remediation periods, high economic costs, difficulties in large-scale applications, and potential ecological risks [15].

Kloepper first coined the term ‘plant growth-promoting rhizobacteria’ (PGPR) in 1978. These beneficial bacteria colonize plant roots and enhance plant growth via multiple direct and indirect pathways. Naturally inhabiting the rhizosphere, these microbes play a pivotal role in improving plant vitality and productivity. PGPR significantly boost their host plants’ tolerance to salt stress via synergistic mechanisms [16]. Direct mechanisms include: (1) regulating ion transport systems to reduce Na^+^ accumulation and augment K^+^ uptake in maize tissues, thereby optimizing Na^+^/K^+^ homeostasis to alleviate ionic toxicity; (2) inducing proline biosynthesis to improve cellular osmoregulation; and (3) markedly increasing antioxidant enzyme activity to strengthen oxidative defense systems [17]. As well, PGPR-secreted phyto-stimulants can directly ameliorate the rhizosphere microenvironment, which can promote the growth and development of roots. Some PGPR are also capable of upregulating salt tolerance-related genes in plants, which enhances their own colonization efficiency and functional performance under stress from salinity [18]. Beyond their direct effects, PGPR can indirectly mitigate salt stress by modulating the rhizosphere’s microbial community structure and nutrient cycling processes, creating a stress-alleviating microenvironment that is beneficial for roots [19].

In recent years, extensive research has revealed that salt-tolerant PGPR in halophyte rhizospheres are ecologically crucial in functioning to help host plants adapt to high-salinity environments [20,21,22,23]. Understandably, these microorganisms have garnered widespread attention due to their inherent salt adaptation capabilities and growth-promoting effects on host plants. Usually, PGPR can be directly isolated and screened from salt-stressed soil ecosystems or from the rhizosphere soil of the plants (especially halophytes) naturally growing in saline environments [24]. Recent work by Lobato-Ureche et al. has demonstrated, for example, that the bacterial strain *Pseudomonas* 42P4 can mitigate the negative impacts of saline stress on pepper plants by enhancing the latter’s morphological, physiological, and biochemical traits under saline conditions [25]. Costa-Gutierrez et al. showed that several *Pseudomonas putida* KT2440 mutant strains (mus-20, mus-42, and EU206) bolster the growth performance of soybean and corn under salt stress conditions, by synergistically improving rhizosphere water conditions through the excessive secretion of extracellular polysaccharides to chelate Na^+^, while simultaneously promoting root development via greater indole-3-acetic acid (IAA) synthesis [26]. Additionally, certain halotolerant rhizobacteria, such as *Pseudomonas pseudoalcaligenes* and *Bacillus subtilis*, can enhance soybean’s salt tolerance by modulating its ion balance, improving its antioxidant enzyme activity, and facilitating its accumulation of osmolytes [27]. As an innovative and very promising bio-enhancement strategy, using PGPR to enhance crop resilience against abiotic stress, in particular that posed by saline–alkali conditions for salt-sensitive crops like maize, has significant advantages over conventional physical and chemical methods. Those advantages include superior environmental compatibility, operational simplicity, exceptional cost-effectiveness, and the potential for real-world, large-scale application [28].

Accordingly, this study had three objectives: (1) to determine the plant growth-promoting (PGP) traits of the *P. fluorescens* G3 strain and its effects on maize seedling growth under salt stress; (2) to identify functional genes associated with salt tolerance and growth promotion in *P. fluorescens* G3, we performed whole-genome sequencing; and (3) to explore the microecological mechanisms of salt resistance using metagenomic sequencing and redundancy analysis (RDA). Hence, this comprehensive study aims both to elucidate the salt-tolerant growth-promoting mechanisms of *P. fluorescens* G3 at multiple scales and to advance the theoretical foundations for developing efficient PGPR-based biological mitigation strategies against salt stress in maize crops.

## 2. Results

### 2.1. Isolation and Identification of Salt-Tolerant Bacteria

**Figure 1:**
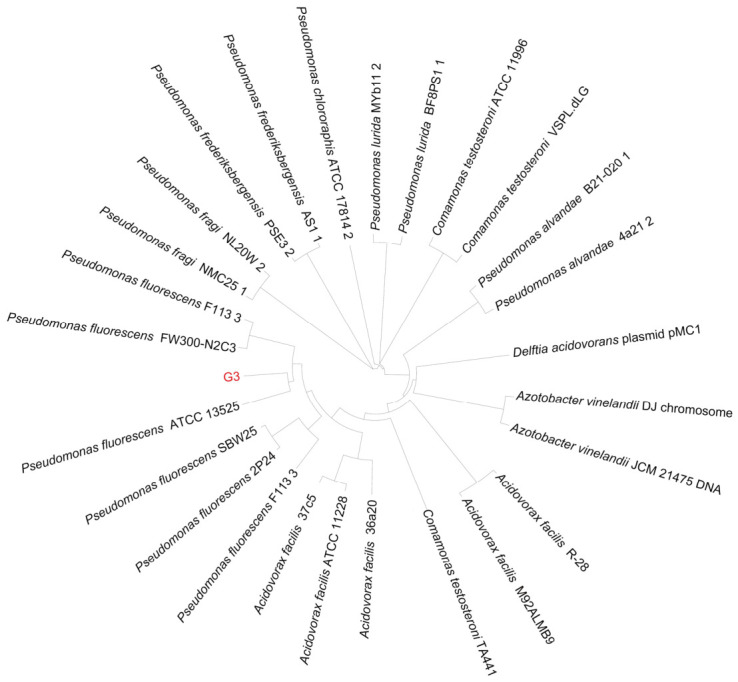
The whole-genome phylogenetic tree of strain G3. The NCBI accession number for this *P. fluorescens* G3 strain is JBUXES010000000.

The target bacterial strain was isolated and purified on LB solid medium supplemented with 850 mmol/L NaCl (pH 8.5). On the LB agar plate containing 850 mmol/L NaCl, this strain could still grow normally, indicating it possessed adequate salt tolerance. Using genomic sequences, a phylogenetic tree was constructed. In it, the strain, *P. fluorescens* G3, was clustered together with *P. fluorescens* ATCC 13525 (Figure 1). The NCBI accession number for this *P. fluorescens* G3 strain is JBUXES010000000.

### 2.2. Determination of P. fluorescens G3’s Tolerance to Varying Salt Stress Concentrations

**Figure 2:**
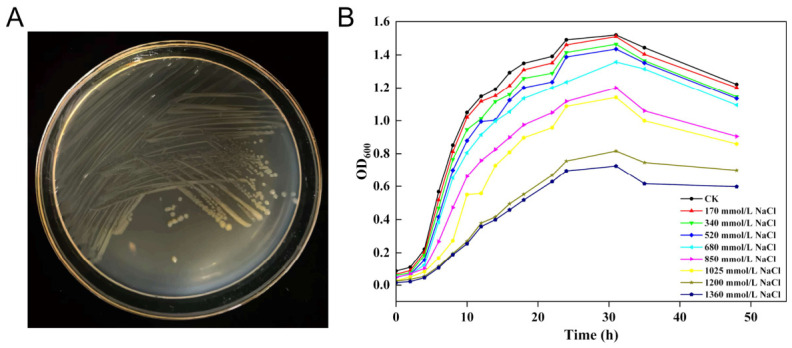
Colony morphology of *P. fluorescens* G3 and salt tolerance of *P. fluorescens* G3. (**A**) Morphology of *P. fluorescens* G3 colonies on LB plates. (**B**) Effect of NaCl concentrations on the growth of *P. fluorescens* G3.

After incubation at 30 °C for 48 h, the colony morphology of *P. fluorescens* G3 was observed on LB agar solid medium. Its colonies appeared creamy white to yellow, being circular, smooth, and translucent (Figure 2A). The salt tolerance of *P. fluorescens* G3 was determined on LB medium (pH 8.5) having NaCl concentrations ranging from 170 to 1360 mM. The colony growth of *P. fluorescens* G3 was recorded at 600 nm after 48 h of incubation at 30 °C. As Figure 2B shows, the growth of *P. fluorescens* G3 showed no significant difference from the CK group at NaCl concentrations of 170–680 mM. When the NaCl concentration rose to 850 and 1020 mM, there was a slight decrease in the growth rate of *P. fluorescens* G3 compared to CK. In response to higher salt stress concentrations (1200 and 1360 mM NaCl), *P. fluorescens* G3 was evidently inhibited but still managed some growth.

### 2.3. PGP (Plant Growth-Promoting) Traits of P. fluorescens G3

**Figure 3:**
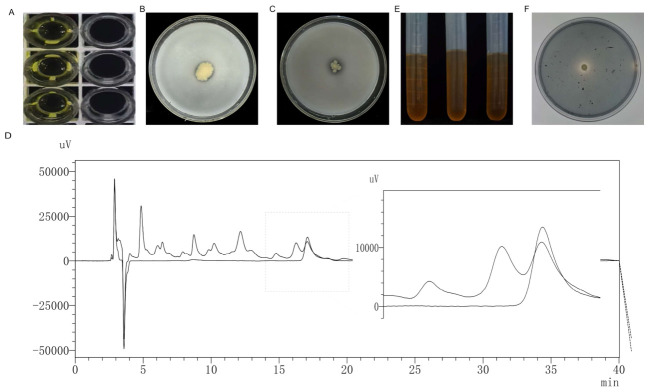
PGP characteristics of *P. fluorescens* G3. (**A**) ACC deaminase 96-well plate results of *P. fluorescens* G3. (**B**) Phosphate-solubilizing results of *P. fluorescens* G3 on MeHKNHa organic phosphorus solid medium plates. (**C**) Phosphate-dissolving results of *P. fluorescens* G3 on MeHKNHa inorganic phosphorus solid medium plates. (**D**) IAA liquid phase results of *P. fluorescens* G3. (**E**) Extracellular polysaccharide secretion results of *P. fluorescens* G3. (**F**) Siderophore production results of *P. fluorescens* G3 on CAS agar medium plates. Note: Data are shown as mean ± SEM (n = 3).

**Figure A1:**
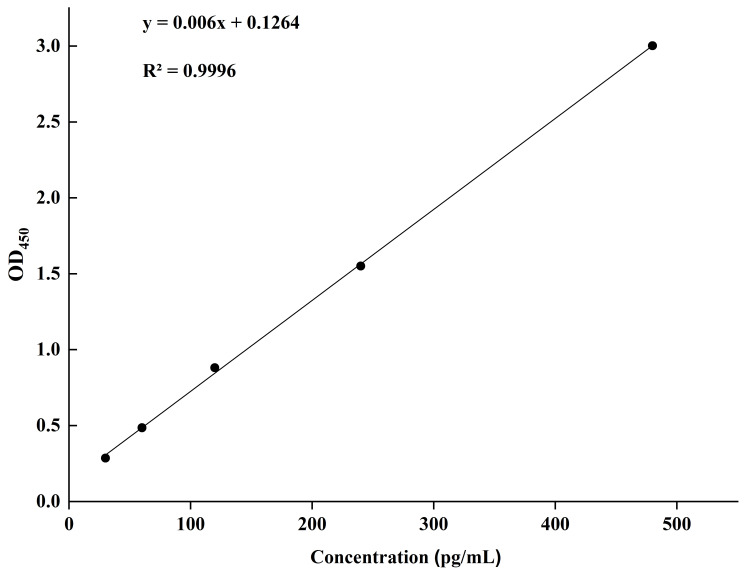
The concentration of the ACC deaminase standard solution.

The purified *P. fluorescens* G3 strain was inoculated into LB liquid medium and fermented for 48 h, followed by testing for its ACC deaminase production capacity. According to the resulting color development of the lysed bacterial suspension obtained with the test kit, *P. fluorescens* G3 produced a pale-yellow color in the reaction, confirming its ability to produce ACC deaminase (Figure 3A). According to the standard curve of ACC deaminase (R^2^ ≥ 0.999; Figure A1), the ACC deaminase production of *P. fluorescens* G3 was calculated to be 105.489 ± 9.17 pg/mL.

On both the MeHKNHa organic and inorganic phosphorus solid medium, *P. fluorescens* G3 produced light-yellow irregular colonies with clear transparent zones around them. After repeated experimental verification, the phosphorus solubilization and phosphorus release abilities of *P. fluorescens* G3 were 2.09 ± 0.10 (15.5/7.4) and 1.82 ± 0.07 (20.2/11.1), respectively (Figure 3B,C).

**Figure A2:**
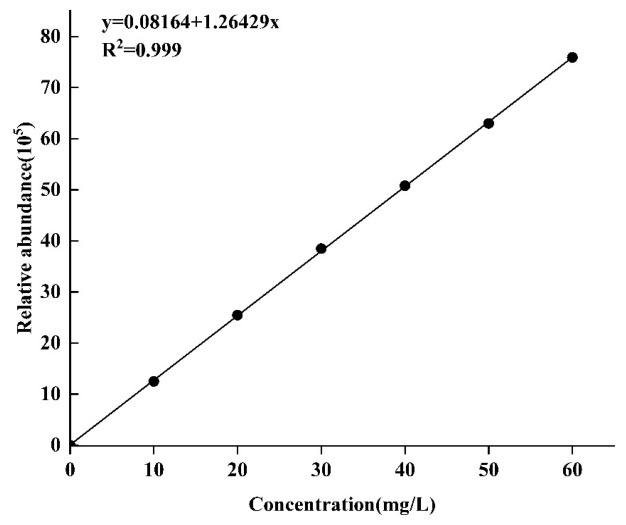
The concentration of the IAA standard solution.

The IAA content of the *P. fluorescens* G3 strain was determined by high-performance liquid chromatography (HPLC). A calibration curve was established with 3-Indoleacetic acid (standard) in the range of 0–100 mg·L^−1^ for precise quantification analysis (Figure A2). The measured IAA production of this strain was 1.686 ± 0.31 mg/L (Figure 3D).

**Figure A3:**
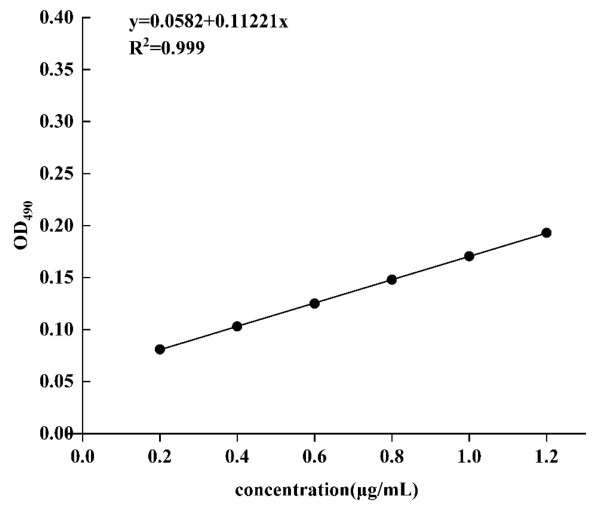
The concentration of the EPS standard solution.

The purified *P. fluorescens* G3 strain was inoculated into LB liquid medium and fermented for 48 h. After centrifugation, the supernatant was collected and reacted with 1 mL of 6–8% phenol and 2.5 mL of concentrated sulfuric acid, resulting in a distinct yellowish-brown color (Figure 3E). Based on their standard curve (Figure A3), the strain’s production of extracellular polysaccharides was calculated to be 10.85 ± 1.68 μg/mL.

Finally, *P. fluorescens* G3 was inoculated onto CAS solid medium and incubated at 30 °C for 3 days. Distinct yellow halos formed around the colonies, indicating the strain’s ability to produce siderophores. The halo diameter (D) to colony diameter (d) (D/d) ratio for siderophore activity was determined to be 2.09 ± 0.37 (11.5/5.5) (Figure 3F).

### 2.4. Effects of P. fluorescens G3 on Maize Growth Under Salt Stress

#### 2.4.1. Germination of Maize Seeds Under Salt Stress

**Figure 4:**
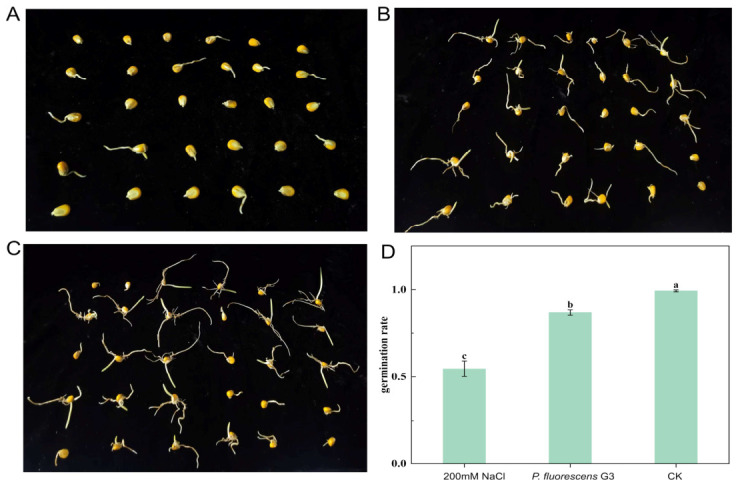
The effect of *P. fluorescens* G3 on maize seed germination. (**A**) The sterile maize seeds under 200 mM NaCl. (**B**) The sterile maize seeds were inoculated with *P. fluorescens* G3 under 200 mM NaCl. (**C**) The sterile maize seeds in sterile water. (**D**) Germination results diagram of maize seeds. Note: Data are shown as mean ± SEM (n = 3). Different lowercase letters (a–c) indicate significant differences at the *p* < 0.05 level.

After 7 days under salt stress (200 mM NaCl), the maize seeds inoculated with *P. fluorescens* G3 had significantly higher germination rates (87% ± 2%) than those treated with sterile water under the same conditions (55% ± 6%) (Figure 4D). Further, the germination rate of the CK group seeds without *P. fluorescens* G3 inoculation under non-stress conditions reached 100% ± 1% (Figure 4D), confirming the sound viability of the tested seeds. This result excludes the possibility that the failed germination of seeds in the stress treatment groups was due to inherent seed quality defects.

#### 2.4.2. Growth of Maize Seedlings Under Salt Stress Conditions

**Figure 5:**
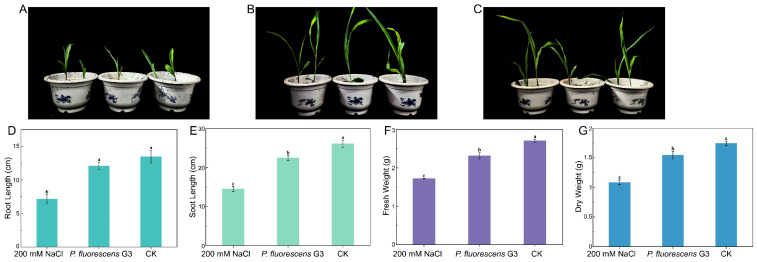
Effect of *P. fluorescens* G3 on maize growth parameters after 21 days of cultivation under 200 mM NaCl conditions. (**A**) The sterile maize seeds under 200 mM NaCl (200 mM NaCl). (**B**) The sterile maize seeds were inoculated with *P. fluorescens* G3 under 200 mM NaCl (*P. fluorescens* G3). (**C**) The sterile maize seeds in sterile water (CK). (**D**) Root length data chart. (**E**) Shoot length data chart. (**F**) Fresh weight data chart. (**G**) Dry weight data chart. Note: Data are shown as mean ± SEM (n = 3). Different lowercase letters (a–c) indicate significant differences at the *p* < 0.05 level.

As Figure 5 shows, compared with the 200 mM NaCl treatment, inoculation with *P. fluorescens* G3 under the same conditions significantly enhanced the growth of maize seedlings. Specifically, their root length and plant height increased by 68.70% ± 8% and 54.80% ± 5%, respectively; their biomass accumulation also significantly improved, with fresh weight and dry weight increasing by 34.60% ± 3% and 42.50% ± 2%, respectively. Notably, all these growth variables showed similar responses between seedlings in the *P. fluorescens* G3 group and those in the CK group (Figure 5). These results confirmed that *P. fluorescens* G3 is able to effectively alleviate the inhibitory impact of salt stress on maize seedling vigor, significantly promoting their growth and development under stress conditions.

#### 2.4.3. Chlorophyll, MDA, and Proline Contents

**Figure 6:**
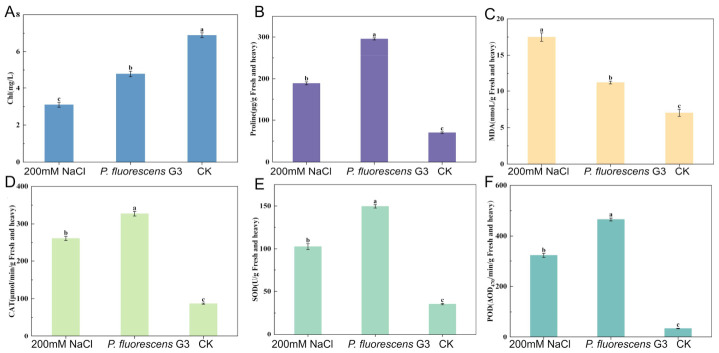
Physiological and biochemical indicators of maize seedlings. (**A**) Chlorophyll content index (Chl). (**B**) Proline content (proline). (**C**) Malondialdehyde content (MDA). (**D**) Catalase content (CAT). (**E**) Superoxide dismutase content (SOD). (**F**) Peroxidase content (POD). Note: Data are shown as mean ± SEM (n = 3). Different lowercase letters (a–c) indicate significant differences at the *p* < 0.05 level.

Maize seedlings inoculated with the *P. fluorescens* G3 strain exhibited a 54.75% ± 8% significant increase in their chlorophyll content in comparison to the 200 mM NaCl treatment group, while being only 30.46% ± 5% lower than the CK group (Figure 6A).

Proline is a key compatible solute and protective molecule that accumulates in plants under stress conditions. As Figure 6B shows, the proline content of maize seedlings inoculated with *P. fluorescens* G3 significantly exceeded that in the 200 mM NaCl treatment group and the CK group, increasing by 56.37% ± 4% and 73.65% ± 2%, respectively.

Conversely, the MDA content of maize seedlings inoculated with *P. fluorescens* G3 was reduced significantly, by 35.76% ± 9%, vis-à-vis the 200 mM NaCl treatment group, though it was still 60.13% ± 6% higher than the CK group (Figure 6C).

#### 2.4.4. Antioxidant Enzyme Activity

This study systematically measured three key antioxidant enzymes-catalase (CAT), superoxide dismutase (SOD), and peroxidase (POD) in maize seedlings inoculated with the endophytic *P. fluorescens* G3 versus non-inoculated controls under salt stress conditions. The enzymatic measurements of maize seedling tissues indicated that, compared to the 200 mM NaCl treatment group, in maize seedlings, the activity of CAT, SOD, and POD was significantly enhanced after inoculation with *P. fluorescens* G3, increasing by 101% ± 10%, 46% ± 6%, and 44% ± 8%, respectively (Figure 6D–F). Under non-stress conditions, moreover, the CK group exhibited extremely low levels of CAT, SOD, and POD activity.

#### 2.4.5. Na^+^ and K^+^ Ions in Plant Tissues

**Figure 7:**
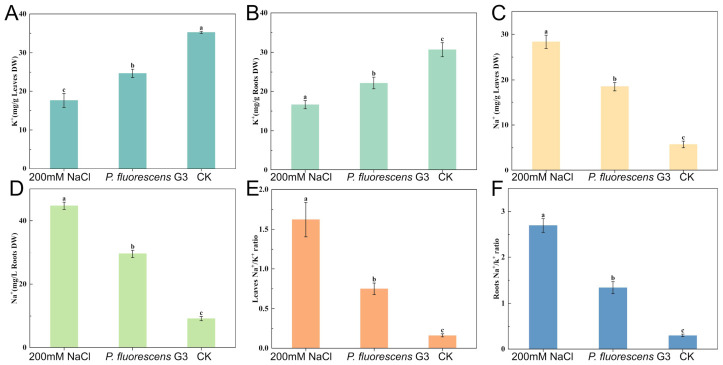
Changes in ion content and ion transport-related genes colonized with or without *P. fluorescens* G3 under normal and salt conditions in maize. (**A**) The potassium ion content in leaves (K^+^ Leaves). (**B**) The potassium ion content in roots (K^+^ Roots). (**C**) The sodium ion content in leaves (Na^+^ Leaves). (**D**) The sodium ion content in roots (Na^+^ Roots). (**E**) Na^+^/K^+^ ratio in leaves. (**F**) Na^+^/K^+^ ratio in roots. Note: Data are shown as mean ± SEM (n = 3). Different lowercase letters (a–c) indicate significant differences at the *p* < 0.05 level.

As shown in Figure 7A–D, the sodium ion content of leaves and roots of maize seedlings inoculated with *P. fluorescens* G3 decreased by 34.90% ± 5% and 33.91% ± 7%, respectively, relative to the 200 mM NaCl treatment group. The CK group under unstressed conditions had an extremely low sodium ion content for leaves or roots.

Compared with the 200 mM NaCl treatment group, the potassium ion content of leaves and roots of maize seedlings inoculated with *P. fluorescens* G3 increased by 40.20% ± 6% and 33.47% ± 4%, respectively. However, it remained 29.95% ± 5% and 27.68% ± 7% lower than that of the CK group’s leaves and roots, respectively (Figure 7A–D).

### 2.5. Genomic Analysis of the P. fluorescens G3 Strain

#### 2.5.1. Main Characteristics of the *P. fluorescens* G3 Genome

**Figure 8:**
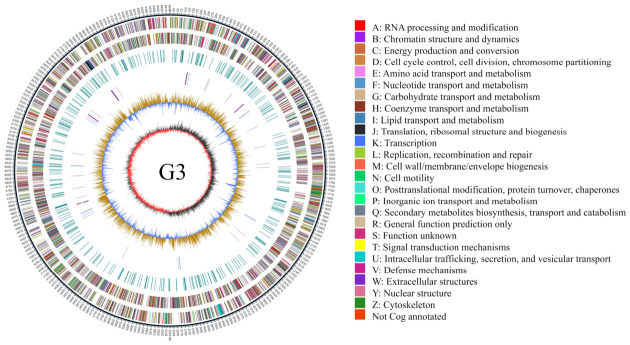
Genome map of *P. fluorescens* G3. The outermost circle represents the genome size scale, with each tick mark indicating 5 kb. The second and third circles represent genes on the positive and negative strands of the genome, respectively, with different colors indicating different COG functional categories. The fourth circle represents repetitive sequences. The fifth circle shows tRNA and rRNA, with blue indicating tRNA and purple representing rRNA. The sixth circle illustrates GC content, where light yellow areas indicate regions with GC content higher than the genome average (the higher the peak, the greater the deviation from the average), and blue areas represent regions with GC content lower than the genome average. The innermost circle shows GC-skew, with dark gray representing regions where G content exceeds C content, and red indicating regions where C content exceeds G content.

The genomic map of *P. fluorescens* G3 was generated using the GC view program. The strain’s complete genome consists of a single circular chromosome with a total length of 6,500,888 bp and a GC content of 61.28%, containing 5519 predicted protein-coding genes, 16 rRNA genes, and 67 tRNA genes (Figure 8).

**Figure 9:**
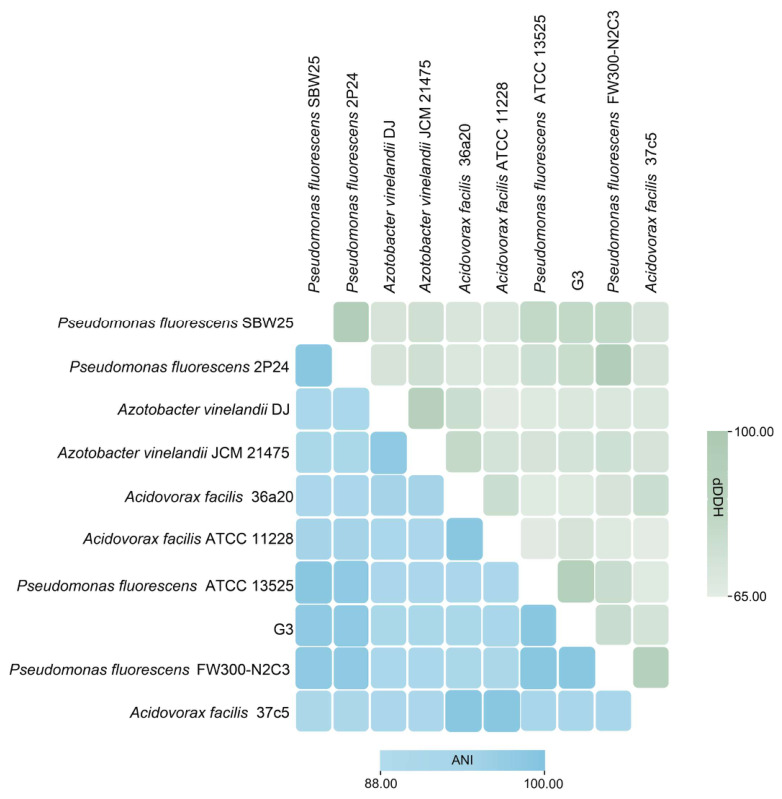
Validation results of ANI and dDDH for *P. fluorescens* G3.

Both ANI (average nucleotide identity) and dDDH (digital DNA-DNA hybridization) analyses are widely used to assess strain similarity based on whole-genome sequences [29]. Those strains having ANI values ≥ 96% and dDDH values ≥ 70% are generally classified as being of the same species [30]. Here, the values between *P. fluorescens* G3 and *P. fluorescens* ATCC 13525 were all above the threshold standards (ANI = 98.2%, dDDH = 80.9%). Thus, the taxonomic identity of the strain investigated in this study was confirmed to be *P. fluorescens* G3 (Figure 9).

#### 2.5.2. Key Genes Related to Salt Tolerance and Plant Growth Promotion in *P. fluorescens* G3

**Figure 10:**
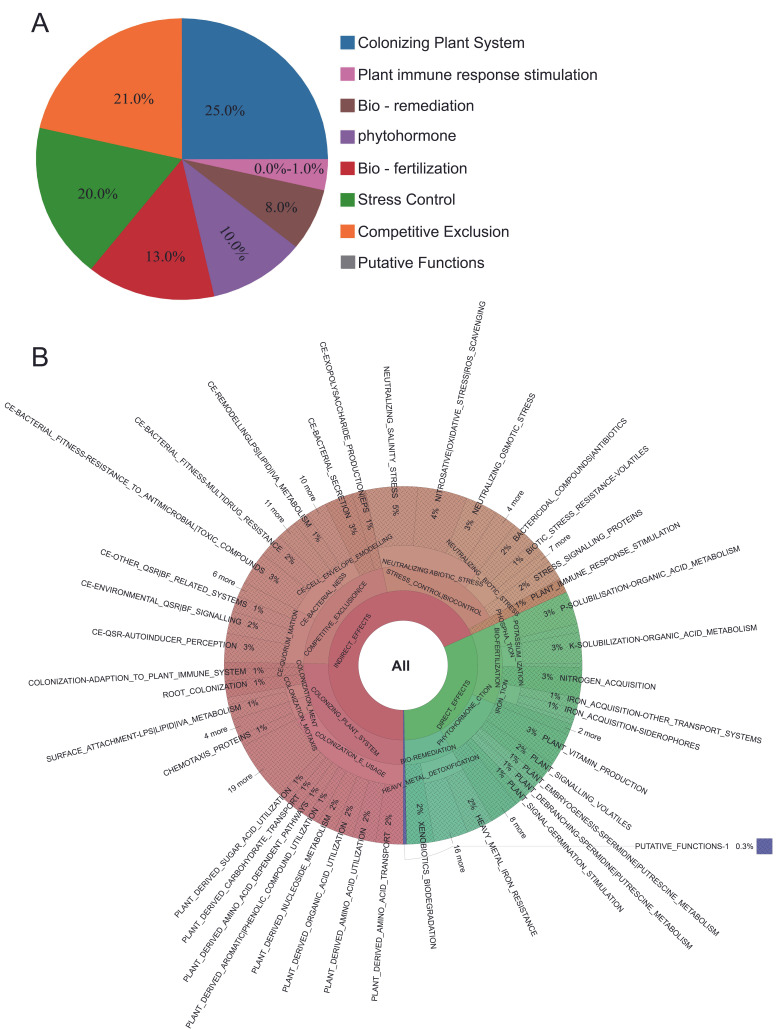
The functional genes of *P. fluorescens* G3 are associated with plant growth-promoting traits. (**A**) The proportion of various functional genes in the genome. (**B**) Function genes that directly or indirectly affect plant growth.

PGPT-Pred is an analytical tool for predicting plant growth-promoting traits in individual bacterial genomes [31]. This revealed that *P. fluorescens* G3 had the following distribution of genes: 25% were associated with host plant-colonization systems, 21% with competitive exclusion, 20% with stress control mechanisms, 13% with bio-fertilization, 10% with phytohormones, 8% with bioremediation, and 0–1% with plant immune response stimulation (Figure 10A,B). The *P. fluorescens* G3 may directly or indirectly promote the growth of host plants via these genes, implying it harbors growth-promoting functions.

Importantly, we found that *P. fluorescens* G3 contains multiple genes related to the synthesis, metabolism, and signal transduction of salt-tolerance pathways. Namely, for synthesis and transport: *proV*, *proW*, and *proP*; for ion homeostasis and stress metabolism: *trkA*, *nhaA*, *sodA*, and *sodB*; and for signal transduction: *envZ* and *ompR* (Table A1).

#### 2.5.3. Expression Levels of Functional Genes in the *P. fluorescens* G3 Strain Under Salt Stress

**Figure 11:**
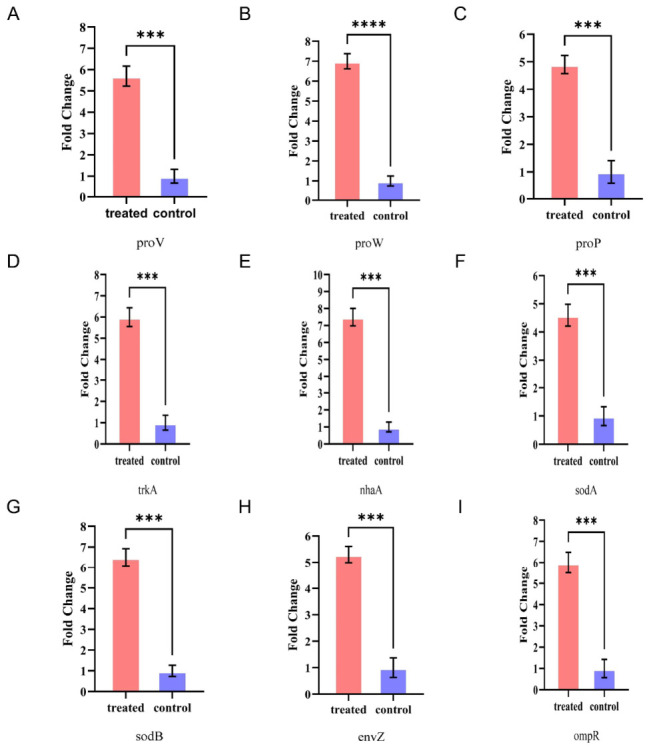
Relative expression levels of (**A**) *proV*. (**B**) *proW*. (**C**) *proP*. (**D**) *trkA*. (**E**) *nhaA*. (**F**) *soda*. (**G**) *sodB*. (**H**) *envZ* and (**I**) *ompR* genes in *P. fluorescens* G3. Bars are the mean ± SE, n = 3. Note: Significant differences from the control group are indicated by *** (*p* ≤ 0.001) or **** (*p* ≤ 0.0001).

The reliability of the *P. fluorescens* G3’s whole-genome dataset was confirmed by RT-qPCR (Table A2), which was used to quantify the mRNA expression levels of genes involved in its salt stress response. As seen in Figure 11, compared with the 200 mM NaCl stress treatment group, the *P. fluorescens* G3-inoculated treatment group underwent significantly upregulated expression of these genes: levels of *proV*, *proW*, and *proP* (synthesis and transport) were upregulated by 2.5-fold, 2.8-fold, and 2.3-fold, respectively; levels of *trkA*, *nhaA*, *sodA*, and *sodB* (ion homeostasis and stress metabolism) were upregulated by 2.6-fold, 2.9-fold, 2.2-fold, and 2.7-fold, respectively; levels of *envZ* and *ompR* (signal transduction regulation) were upregulated by 2.4-fold and 2.6-fold, respectively (Table A3). Altogether, these results suggested that these upregulated genes could play an active role in how *P. fluorescens* G3 responds to 200 mM NaCl stress.

### 2.6. Effects of P. fluorescens G3 on Maize Rhizosphere’s Microbial Community Under Salt Stress

#### 2.6.1. Microbial Taxonomic Diversity and Composition

**Figure A4:**
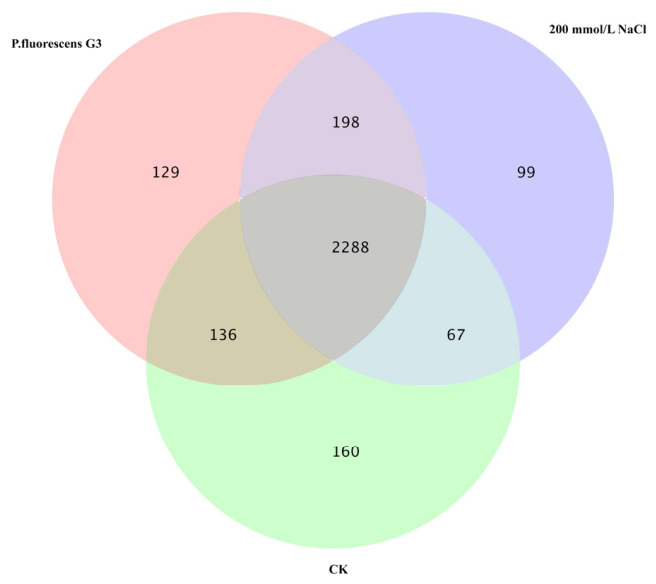
Venn diagram analysis of different treatment groups (values indicate intersection sample size).

According to the Venn diagram (Figure A4), the three different treatment groups (*P. fluorescens* G3-inoculated + 200 mM NaCl salt stress; non-inoculated + 200 mM NaCl salt stress; and lacking inoculation with *P. fluorescens* G3 and salt stress) shared 2288 OTUs that presumably maintained the integrity of the rhizosphere community. The group with *P. fluorescens* G3- inoculation coupled to salt stress had 129 unique OTUs, showing greater similarity to the non-inoculated control (136 shared OTUs) than the non-inoculated group under salt stress (67 shared OTUs).

**Figure 12:**
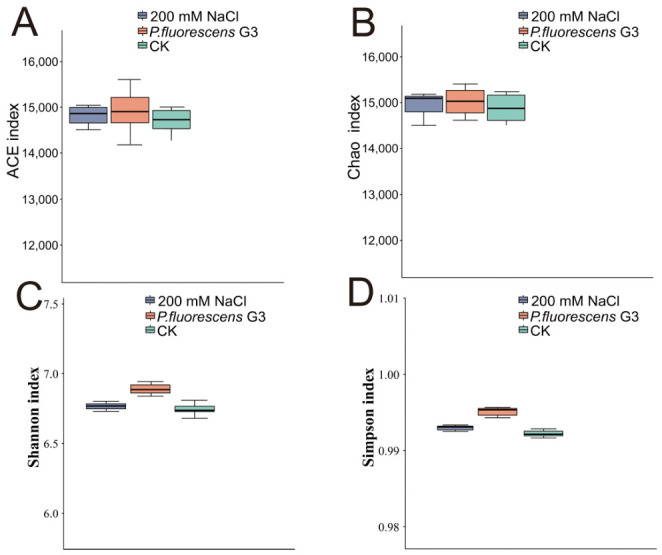
Analysis of α-diversity in maize rhizosphere soil. (**A**): Distribution of ACE index of microbial community in soil/samples under different treatments. (**B**): Distribution of Chao index of microbial community in soil/samples under different treatments. (**C**): Distribution of Shannon index of microbial community in soil/samples under different treatments. (**D**): Distribution of Simpson index of microbial community in soil/samples under different treatments.

**Figure 13:**
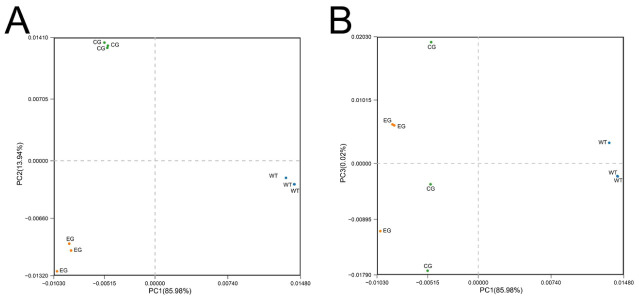
Analysis of principal component analysis in maize rhizosphere soil. Note: WT: CK. EG: *P. fluorescens* G3. CG: 200 mM NaCl. (**A**): PC1–PC2 ordination plot of principal coordinate analysis for microbial community composition in different experimental groups. (**B**): PC1–PC3 ordination plot of principal coordinate analysis for microbial community composition in different experimental groups.

Alpha diversity reflects the taxonomic richness and diversity of individual samples. When compared with the CK and 200 mM NaCl groups, the *P. fluorescens* G3 treatments showed higher ACE and Chao1 index values as well as higher Shannon and Simpson index values (Figure 12). To better compare differences in community composition, we conducted a PCA. As Figure 13A shows, the bacterial community of the *P. fluorescens* G3 treatment separated from that of the CK and 200 mM NaCl treatment along the first and second principal components, respectively (PC1 and PC2; Figure 13B). Together, PC1 and PC2 explained nearly all the variability (ca. 99% = 85.98% and 13.94%, respectively) present in the bacterial community dataset (Figure 13A).

**Figure 14:**
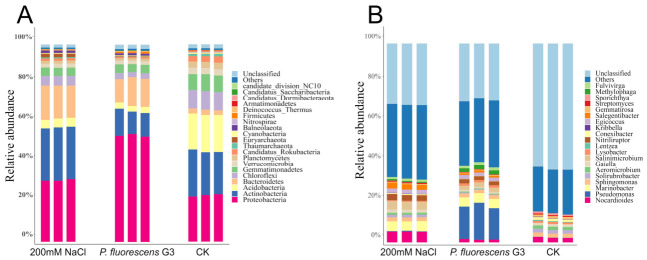
Analysis of bacterial community composition in maize rhizosphere soil. (**A**) Relative abundance of different bacterial phyla within rhizosphere communities in consortia-treated and control soils. (**B**) Relative abundance of different bacterial genera within rhizosphere communities in consortia-treated and control soils.

To further analyze the changes in rhizosphere microbial community under the different treatments, we selected the 20 most abundant taxa at both the phylum and genus levels (Figure 14). *Proteobacteria*, followed by *Actinobacteria*, *Acidobacteria*, *Bacteroidetes*, *Chloroflexi*, and *Gemmatimonadetes*, were the six most abundant bacterial phyla across all samples, together accounting for >80% of all the classified sequences. Among the most abundant phyla, the relative abundance of *Chloroflexi* was lowest while that of *Proteobacteria* was highest in the *P. fluorescens* G3 treatment vis-à-vis the CK and 200 mM NaCl treatment (Figure 14A). Likewise, among the most abundant genera, the relative abundance of *Solirubrobacter* was lowest while that of *Pseudomonas* was highest in the *P. fluorescens* G3 treatment (Figure 14B).

#### 2.6.2. Microbial Functioning in the Rhizosphere Community

**Figure 15:**
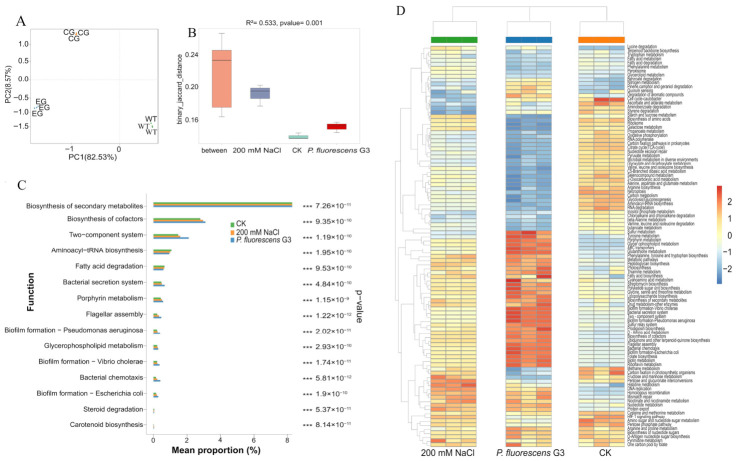
Differential gene expression analysis. (**A**) Principal component analysis of functional abundance. Note: WT: CK (water). EG: *P. fluorescens* G3. CG: 200 mM NaCl. (**B**) PERMANOVA/ANOSIM analysis. (**C**) Horizontal bar chart. Note: The leftmost is the pathway name, with the x-axis representing the average proportion and the y-axis representing the functional names. The rightmost column shows the actual *p*-values (*** for *p* ≤ 0.0001). By default, the top 15 entries are selected after sorting by p-value in descending order, followed by sorting by abundance from highest to lowest. (**D**) Heatmap of differentially expressed gene abundance.

To better compare functional gene differences across taxa in the rhizosphere microbial community, we performed principal component analysis (PCA) of functional abundance profiles. As illustrated in Figure 15A, in this respect, the *P. fluorescens* G3 treatment lay apart from the CK along PC1, as well as the 200 mM NaCl treatment along PC2. Together, both components explained ca. 91% (82.53% and 8.57%, respectively) of the existing variability in the bacterial community dataset (Figure 15A).

The binary Jaccard distance bar chart quantifies the average dissimilarity among samples. When used, we obtained an R^2^ = 0.533 (explaining 53.3% of total variance) and a highly significant *p*-value (≤0.001), indicating extreme differences both between and within the treatment groups (Figure 15B). The “between” bar (representing dissimilarity across different treatment groups) had the highest binary Jaccard distance (≈0.24), being markedly greater than all within-group values. This finding confirmed that treatment conditions were the primary driver of overall sample differences.

Regarding within-group variation, among the three treatment/control groups, the 200 mM NaCl group exhibited the highest within-group dissimilarity (≈0.20), this reflecting pronounced heterogeneity among samples under high salt stress alone. In contrast, the *P. fluorescens* G3 treatment group displayed much lower within-group variation (≈0.16), this being statistically less than that of the 200 mM NaCl group. This result indicated that inoculation with *P. fluorescens* G3 reduced sample heterogeneity under high salt stress, improving the response stability to the treatment. The CK group had the lowest within-group dissimilarity (≈0.14), as expected for a non-stressed, stable system (Figure 15B).

As Figure 15C shows, the horizontal bar chart lists 15 different key pathways that relate most to salt stress adaptation in bacteria. Among them, after treatment with *P. fluorescens* G3, the following pathways showed significant increases: biosynthesis of secondary metabolites, biosynthesis of cofactors, two-component system, bacterial secretion system, porphyrin metabolism, flagellar assembly, biofilm formation-*Pseudomonas* aeruginosa, and bacterial chemotaxis. Moreover, in the differential functional gene abundance heatmap, we found that these eight pathways were all upregulated, having stable and consistent patterns (Figure 15D).

#### 2.6.3. Microbial Community and Environmental Factor Correlations

**Figure 16:**
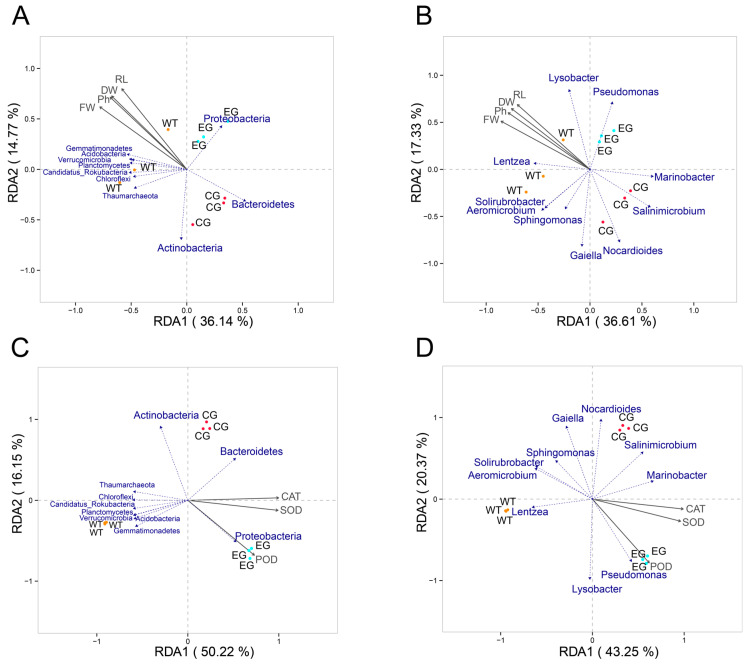
Redundancy analysis (RDA) biplots based on the relative abundance of bacterial phyla and genus levels, plant growth indicators (**A**,**B**), and antioxidant enzyme activity (**C**,**D**). Note: WT: CK, EG: *P. fluorescens* G3, CG: 200 mM NaCl. FW: Fresh weight. DW: Dry weight. Ph: Plant height. RL: Root length.

The RDA was based on the selected physicochemical and biochemical indicators (plant physiological indicators, antioxidant enzyme activity) and the top-10 taxa abundances at the phylum and genus levels (Figure 16). The resulting RDA diagram depicts the influence of plant physiological indicators upon taxa distribution at both taxonomic levels considered (Figure 16A,B). This revealed that plant physiological indicators were strongly associated with the CK, yet they had weak positive relationships with the *P. fluorescens* G3 treatment, but strong negative ones with the 200 mM NaCl treatment. Among the examined physiological indicators, fresh weight (FW) emerged as the most critical environmental factor, followed by plant height (Ph), dry weight (DW), and root length (RL). In Figure 16A, the first component (RDA1) explained 36.14% of the total variation in that data, separating the *P. fluorescens* G3 and 200 mM NaCl treatments from the CK, whereas the second component (RDA2) explained just 14.77% of that total variation, along which the *P. fluorescens* G3 treatment and the 200 mM NaCl treatment were nonetheless segregated.

The bacterial phyla most closely associated with the CK included *Gemmatimonadetes*, *Verrucomicrobia*, *Planctomycetes*, *Candidatus Rokubacteria*, *Chloroflexi*, and *Thaumarchaeota*. The phyla associated most with the *P. fluorescens* G3 treatment were *Proteobacteria*, while *Bacteroidetes* and *Actinobacteria* were tied more to the 200 mM NaCl treatment. In Figure 16B, the first component (RDA1) explained 36.61% of the total variation in that data, separating the *P. fluorescens* G3 and 200 mM NaCl treatments from the CK, while the second component (RDA2) explained just 17.33% of the total variation but still segregated the *P. fluorescens* G3 and 200 mM NaCl treatments. The genera that associated significantly with the CK were *Lentzea* and *Lysobacter*, whereas only *Pseudomonas* did so with the *P. fluorescens* G3 treatment, while *Marinobacter*, *Salinimicrobium*, and *Nocardioides* all did so with the 200 mM NaCl treatment. The corresponding RDA diagram illustrates the influence of antioxidant enzyme activity upon taxa distribution at both phylum and genus levels (Figure 16C,D). Evidently, antioxidant enzyme activity exhibited a strong positive association with the *P. fluorescens* G3 treatment, weak positive relationships with the 200 mM NaCl treatment, and a strong negative association with the CK. Among the antioxidant enzymes, the activity of POD was the most decisive environmental factor, followed by that of SOD, then CAT. In Figure 16C, RDA1 explained 50.22% of that data’s total variation, separating the *P. fluorescens* G3 and 200 mM NaCl treatments from the CK; although RDA2 explained just 16.15% of the total variation, this was sufficient to split the *P. fluorescens* G3 and 200 mM NaCl treatments. The CK-related bacterial phyla included *Candidatus*, *Rokubacteria*, *Actinobacteria*, *Gemmatimonadetes*, *Thaumarchaeota*, and *Chloroflexi*. The *P. fluorescens* G3 treatment-related bacterial phyla were limited to one, *Proteobacteria*, whereas the 200 mM NaCl treatment-related bacterial phyla mainly comprised *Bacteroidetes* and *Actinobacteria*. Similarly, in Figure 16D, it can be seen that RDA1 explained 43.25% of the total variation in this data, likewise separating the *P. fluorescens* G3 and 200 mM NaCl treatments from the CK, with RDA2 explaining another 20.37% of the total variation and separating the *P. fluorescens* G3 and 200 mM NaCl treatments. The genera significantly associated with the CK, the *P. fluorescens* G3 treatment, and the 200 mM NaCl treatment were *Lentzea*, *Pseudomonas*, and *Salinimicrobium*, respectively.

## 3. Discussion

This study isolated salt-tolerant *P. fluorescens* G3 from maize rhizosphere soil in saline–alkali soils (Lanzhou, Gansu Province, China). This *P. fluorescens* G3 strain was selected because of its impressive ACC deaminase activity. Genomic similarity analysis further confirmed its species classification as *P. fluorescens*. Through integrated physiological-biochemical characterization, genomic analysis, and plant growth assays, our work shows that *P. fluorescens* G3 has robust salt-tolerance mechanisms, plant growth-promoting (PGP) traits, and the capacity to mitigate the salt stress incurred by maize seedlings. Experimental results demonstrate that *P. fluorescens* G3 maintains stable growth in the face of substantial salinity (spanning 170–1360 mM NaCl) and alkaline conditions (pH 8.5). Furthermore, it is effective at alleviating salt stress in maize seedlings [32,33,34] by regulating the host plant’s physiological status and enhancing its rhizosphere microbial community via multiple PGP mechanisms, including phosphate solubilization, indole-3-acetic acid (IAA) production, ACC deaminase activity, and extracellular polysaccharide (EPS) synthesis. Collectively, these findings highlight the potent biological role of *P. fluorescens* G3 in plant–microbe interactions under stress conditions and underscore its considerable potential for practical applications in developing microbial inoculants to improve crop resilience in saline–alkali agriculture.

### 3.1. Salt-Tolerance Mechanisms and Adaptability of P. fluorescens G3

The saline–alkali soils of Gansu Province are characterized by very high salinity (850–1360 mM NaCl) and alkalinity (pH 8–9). The strain *P. fluorescens* G3 isolated from that environment exhibits significant salt tolerance. It maintained robust colony growth (OD_600_ = 1.17) at 850 mM NaCl and remained viable even at 1360 mM NaCl [35]. Our genomic analysis detected multiple functional genes associated with salt tolerance in the *P. fluorescens* G3 genome. Their expression likely underpins its salt tolerance through several key pathways. (1) Ion homeostasis [36]: The genes *trkA* and *nbaA*, encoding ion transporters, facilitate K^+^ uptake and Na^+^ extrusion, which helps to maintain cytoplasmic Na^+^/K^+^ homeostasis. (2) Osmoprotectant synthesis [37]: The *proV* and *proW* genes are involved in the synthesis of betaine, a compatible solute that reduces cellular osmotic stress and mitigates the damage to proteins and membranes caused by salt ions. Osmosensing and regulation [38]: The *envZ* and *ompR* genes encode a key two-component system which senses osmotic changes and regulates the expression of outer membrane porins, thus figuring prominently in the environmental adaptation of bacteria. This confirms that the salt tolerance ability of *P. fluorescens* G3, as mediated by these conserved molecular mechanisms, provides a crucial foundation for its adaptation and survival in saline–alkali soil environments [39].

### 3.2. Plant Growth-Promoting (PGP) Traits of Microorganisms That Enhance Host Plant Growth Under Stress Conditions

We find that *P. fluorescens* G3 harbors significant PGP traits relevant to alleviating salt stress in maize. First, the formation of distinct transparent zones on MeHKNHa solid medium supplemented with organic and inorganic phosphorus sources [40], with D/d ratios of 2.09 (15.5/7.4) and 1.82 (20.2/11.1) respectively, indicates the strain’s efficient solubilization of insoluble phosphorus. This capability is vital in saline–alkali soils, where phosphorus readily precipitates with Ca^2+^, leading to a phosphorus deficiency in plants. By increasing the available phosphorus in soil, *P. fluorescens* G3 alleviates that nutrient’s deficiency, thereby promoting maize’s root growth and dry matter accumulation.

The studied strain produced IAA (1.686 mg/L) [41]. Although its IAA production may not be ideal, this does not entirely preclude its potential to promote plant growth through multiple mechanisms in the rhizosphere microenvironment. Numerous studies have shown that PGPR’s growth-promoting effects often result from the combined action of various mechanisms. While *P. fluorescens* G3’s IAA production may not reach the levels of certain high-efficiency strains, as part of a multi-mechanism plant growth-promoting strain, it may still contribute positively to maize growth in combination with other growth-promoting characteristics. The *P. fluorescens* G3 strain secreted ACC deaminase (105.489 pg/mL). This enzyme degrades 1-aminocyclopropane-1-carboxylic acid (ACC) [42], the immediate precursor of ethylene in the roots of many food crops. Salt stress raised the ACC levels in plants, leading to excessive ethylene synthesis that inhibits their root growth and cell division. By reducing the ACC concentration and subsequent ethylene production, the ACC deaminase supplied by *P. fluorescens* G3 can contribute to ameliorating the stress-induced growth inhibition of maize.

This strain also secreted EPS (10.853 μg/mL), a high-molecular-weight polymer that forms a protective bacterial biofilm on root surfaces [43]. This layer reduces the adsorption and uptake of detrimental ions (e.g., Na^+^) and enhances water retention in the rhizosphere, thereby mitigating osmotic stress. Consistent with this mechanism, the EPS content of rhizosphere soil from maize seedlings inoculated with *P. fluorescens* G3 is significantly greater than in non-inoculated controls, suggesting its key role in limiting Na^+^ accumulation.

### 3.3. Improvement in Maize Seedlings’ Physiological Status and Salt Tolerance

Under salt stress, inoculation with *P. fluorescens* G3 significantly boosted the activities of powerful antioxidant enzymes in maize seedlings: CAT by 101%, SOD by 46%, and POD by 44%. Concurrently, their MDA content, a marker of lipid peroxidation, decreased by 35.76% [44]. Our genomic analysis identified *sodA* and *sodB* genes within *P. fluorescens* G3 that encode SOD enzymes able to directly scavenge for damaging superoxide radicals, which likely contributed to the observed reduction in MDA accumulation [45].

Maize seedlings inoculated with *P. fluorescens* G3 benefitted from a 56.37% increase in their proline content while under salt stress [46]. This strain’s bacterial genome harbors *proV* and *proW* genes responsible for synthesizing the compatible solute betaine. This bacterial betaine production, combined with the ACC deaminase activity (105.489 pg/mL) of *P. fluorescens* G3, can synergistically mitigate cellular osmotic stress. Further, ACC deaminase, by degrading the ethylene precursor ACC and reducing ethylene levels, indirectly promotes proline synthesis in the host plant, enhancing its overall cellular osmotic regulation capacity.

Inoculation with *P. fluorescens* G3 significantly altered the ion profiles in salt-stressed maize seedlings: their sodium ion (Na^+^) content fell by 34.90% in leaves and 33.91% in roots, while potassium ion (K^+^) content rose by 40.20% in leaves and 33.47% in roots. Our genomic analysis uncovered the presence of *trkA*, it encoding a high-affinity K^+^ transporter facilitating K^+^ uptake, as well as *nhaA*, which encodes a Na^+^/H^+^ antiporter that drives Na^+^ efflux [47]. These genetic findings provide a basis for the direct modulation of the ionic environment by *P. fluorescens* G3. Combined with its other PGP traits and community effects discussed below, this contributes to the overall optimization of Na+/K+ homeostasis in its host plant.

Salt stress helps bacteria perceive stress and initiate their own stress resistance responses. Bacteria assist plants through various means (which may include secreting proline, producing other metabolites, triggering plant systemic resistance, etc.). With bacterial assistance, the plant’s own stress-resistant physiology (including potentially enhanced proline synthesis) operates more effectively, ultimately manifested as increased proline content in maize and stress mitigation. Therefore, the upregulation of bacterial genes serves as an indispensable ‘link’ evidence in this interaction chain, indicating that bacteria possess the potential capacity to enhance proline synthesis under stress conditions. This provides mechanistic plausibility for explaining the positive outcomes of the entire system.

Based on the above plant physiological and genomic evidence, we propose that *P. fluorescens* G3 alleviates salt stress by directly or indirectly optimizing the ion homeostasis of host maize. Direct action: The high-affinity potassium transporter (*trkA*) and sodium/proton antiporter (*nhaA*) encoded in *P. fluorescens* G3 genome provide a molecular basis for its active uptake of K^+^ and efflux of Na^+^ in the rhizosphere microenvironment. This direct ion regulation activity may effectively alter the ion availability in roots and leaves, reducing Na^+^ influx into plant tissues and increasing K^+^ uptake at the source. This directly contributes to the observed decrease in Na^+^ and increase in K^+^ in plant tissues. Indirect action: *P. fluorescens* G3 systematically enhances the plant’s intrinsic capacity to maintain ion homeostasis by secreting ACC deaminase to reduce stress-induced ethylene and producing IAA to promote root growth. This may include inducing plant intrinsic ion transport pathways such as the SOS system. More importantly, as subsequent analyses show, *P. fluorescens* G3 colonization significantly reshapes the structure and function of the rhizosphere microbial community, enriching synergistic bacterial populations. This indirectly supports plant ion balance and stress tolerance through the reinforcement of overall microbiome functionality. Therefore, the improvement in ion content in maize seedlings is not attributed to a single mechanism, but rather results from the combined effects of *P. fluorescens* G3’s direct ion regulation, systemic induction in plants, and synergistic reconstruction of the beneficial rhizosphere microbiome. This provides a reasonable mechanistic explanation for how a single probiotic strain can produce multiple systemic benefits.

### 3.4. Mechanisms by Which P. fluorescens G3 Alleviates Salt Stress Through Functional Gene Regulation of the Rhizosphere Microbiome

Metagenomic analysis revealed that, under salt stress, inoculation with *P. fluorescens* G3 significantly enriched various functional pathways within the rhizosphere microbiome known to be associated with stress adaptation. This enrichment promoted key physiological processes in maize seedlings, demonstrating strong plant growth-promoting effects, including ion homeostasis, antioxidant defense, osmotic regulation, and enhanced biomass accumulation and photosynthetic efficiency.

Inoculation with the *P. fluorescens* G3 strain also significantly promoted maize seedlings’ growth under salt stress, increasing their root length by 68.7%, plant height by 54.8%, fresh weight by 34.6%, and dry weight by 42.5%. These benefits are facilitated by the upregulation of *P. fluorescens* G3 genes involved in bacterial chemotaxis and flagellar assembly, both enabling targeted root colonization. Following the host’s colonization, the bacterial secretion of IAA stimulates the development of roots and expands their nutrient-absorptive surface area. In tandem, the upregulation of bacterial secretion system genes in G3 potentially facilitates the delivery of effector molecules, optimizing plant–microbe interactions and contributing to its PGP traits.

We found that *P. fluorescens* G3 inoculation significantly reduced the Na^+^ content of maize leaves and roots by 34.9% and 33.9%, respectively. The upregulation of biofilm formation genes in the strain likely enhances the host plant’s root colonization density, forming a protective barrier that decreases Na^+^ infiltration and directly alleviates osmotic stress. Moreover, the upregulation of two-component system genes would have promoted the synthesis of compatible solutes (e.g., glycine betaine) through the regulation of osmotic stress-responsive genes. These bacterial solutes can work synergistically with the proline accumulated by plants to maintain cellular osmotic balance.

Inoculated maize seedlings exhibited significantly enhanced antioxidant enzyme activity under salt stress, with increases of 101% for catalase (CAT), 46% for superoxide dismutase (SOD), and 44% for peroxidase (POD), accompanied by a 35.8% decrease in their malondialdehyde (MDA) content. The upregulation of genes for the biosynthesis of cofactors in the rhizosphere microbiome may bolster CAT and POD activity there by supplying essential prosthetic groups (e.g., heme for CAT). Similarly, the provision of metal cofactors (Fe, Mn, Cu, Zn) via this pathway is essential for optimal SOD activity.

Inoculation with the *P. fluorescens* G3 strain increased the photosynthetic pigment content of maize seedlings by 54.8%. This beneficial change may be attributed, in part, to the *P. fluorescens* G3-induced promotion of secondary metabolite biosynthesis pathways, which would help mitigate oxidative stress and thereby contribute to chlorophyll stability.

### 3.5. Rhizosphere Microbial Community’s Regulation

Analysis of the rhizosphere microbial community revealed that its composition was significantly altered by inoculation with *P. fluorescens* G3 under salt stress. Specifically, it increased the relative abundance of γ-*Proteobacteria*, establishing Pseudomonas as the dominant genus, while reducing the prevalence of Actinobacteria populations. This shift may be due to competitive interactions and niche modification induced by the introduction of *P. fluorescens* G3 into the community.

As a member of the γ-*Proteobacteria* class, the successful colonization of *P. fluorescens* G3 in the rhizosphere directly contributed to the increased Proteobacteria abundance. The species-level analysis confirmed that *P. fluorescens* was exclusively detected in the inoculated treatments. As well, metabolites produced by *P. fluorescens* G3, including extracellular polysaccharides (EPS), indole-3-acetic acid (IAA), and potential antibiotics, likely inhibited the growth of competing microorganisms such as actinomycetes (*Actinobacteria*). Simultaneously, these metabolites may have stimulated the proliferation of other beneficial plant growth-promoting rhizobacteria (PGPR) within the *Proteobacteria* phylum. Finally, we find that *P. fluorescens* G3 improved key rhizosphere soil physicochemical properties (e.g., nutrient availability, osmotic balance), creating conditions more favorable for salt-tolerant microorganisms, predominantly those within the *Proteobacteria* phylum over *Actinobacteria*.

It is important to bear in mind that synergistic interactions between *P. fluorescens* G3 and co-enriched *Proteobacteria* PGPR could have amplified any plant growth-promoting effects. Correspondingly, a reduction in the *Actinobacteria* abundance might diminish the production of particular growth-inhibiting metabolites (e.g., certain antibiotics), fostering a rhizosphere microenvironment more conducive to maize growth, development, and reproduction.

A redundancy analysis linking the microbial community profiles with plant physiological parameters showed that the community shift explained a substantial proportion (66.37%) of the variance in plant biomass and stress indicators. These results suggest that the plant growth promotion under salt stress was not directly due to the abundance of *P. fluorescens* G3 perse, but was mediated by the *P. fluorescens* G3-induced reconstitution of a cooperative rhizosphere microbiome, which collectively enhanced nutrient acquisition and stress tolerance for the host plant. This strongly supports the concept that the plant growth promotion is mediated not solely by the direct actions of *P. fluorescens* G3, but significantly through the *P. fluorescens* G3-induced reconstitution of a cooperative rhizosphere microbiome, highlighting the synergy between the ‘direct’ and ‘community-mediated indirect’ mechanisms.

### 3.6. Correlation Between P. fluorescens G3 Physiological Phenotype and Maize Salt Resistance

First, the core physiological traits of *P. fluorescens* G3 directly address the key limiting factors of salt stress. *P. fluorescens* G3-secreted ACC deaminase degrades ACC, the ethylene precursor abundantly synthesized by plants under stress, thereby alleviating senescence and oxidative damage caused by ethylene accumulation. This mechanism likely explains the significant reduction in MDA content observed in maize leaves after *P. fluorescens* G3 inoculation. Meanwhile, the IAA secreted by *P. fluorescens* G3 promotes maize root development, which enhances the plant’s ability to acquire water and nutrients under salt stress, thereby laying the foundation for maintaining physiological functions. Furthermore, the exopolysaccharides produced by *P. fluorescens* G3 not only facilitate its own colonization in the rhizosphere but may also indirectly mitigate ionic toxicity and osmotic stress by chelating Na^+^ and improving water retention in the rhizosphere microenvironment.

Secondly, the physiological traits of *P. fluorescens* G3 enhance stress resistance by modulating the intrinsic physiological status of plants. *P. fluorescens* G3’s phosphate-solubilizing and siderophore-producing capabilities improve plant utilization efficiency of essential elements (e.g., phosphorus and iron) under salt stress, thereby supporting enhanced metabolism (such as photosynthesis and increased chlorophyll content) and the synthesis of antioxidant compounds.

### 3.7. Multi-Level Mechanisms Underlying Salt Tolerance Enhanced by P. fluorescens G3

At the molecular level, *P. fluorescens* G3 possesses intrinsic salt stress-responsive and growth-promoting genes. Genomic analyses confirm it carries *trkA* (K^+^ uptake), *nhaA* (Na^+^ efflux), and other genes that directly optimize rhizosphere Na^+^/K^+^ balance. Its genome also harbors key PGP trait genes for IAA synthesis, ACC deaminase secretion, and EPS production. These are not isolated functions but form the molecular basis for systemic plant regulation (e.g., root development promotion, stress ethylene reduction, and physical barrier formation).

Plant physiological responses: Improved ion homeostasis with significantly reduced Na^+^ and increased K^+^ content in leaves and roots; activated antioxidant system with substantially enhanced SOD, POD, and CAT activities alongside decreased MDA levels; strengthened osmotic adjustment through elevated endogenous proline accumulation; and growth recovery evidenced by markedly improved root length, plant height, and biomass. These physiological changes serve as the critical bridge linking bacterial functions to the ultimate salt-tolerant phenotype in plants.

Rhizosphere ecological restructuring: Significant enrichment of beneficial taxa such as *Pseudomonas* and *Methylophaga*; metagenomic analyses reveal that *P. fluorescens* G3 introduction systematically upregulates the functional potential of the entire rhizosphere microbiome in ion homeostasis, antioxidant defense, and osmotic adjustment; redundancy analysis (RDA) demonstrates that these *P. fluorescens* G3-driven microbial community shifts explain 66.37% of the variation in plant biomass and stress indicators. This robustly proves that *P. fluorescens* G3’s benefits are primarily mediated by reconstructing a more resilient and functional rhizosphere microbiome.

In summary, the mechanism by which *P. fluorescens* G3 enhances maize salt tolerance is multifaceted: it first utilizes its intrinsic capacities to directly improve the rhizosphere environment and activate primary plant responses; more critically, it amplifies these bacterial effects into systemic resilience of the entire rhizosphere ecosystem by reshaping the microbial ecology to favor a beneficial community.

## 4. Materials and Methods

### 4.1. Isolation and Identification of Salt-Tolerant Rhizosphere Bacteria

Soil samples from the rhizosphere of cropped maize plants were collected in the salt area of Lanzhou New District, Lanzhou City, in Gansu Province, China (36°17′07″ N, 103°35′33″ E). From their diluted soil suspension, we isolated salt-tolerant bacteria. These colonies were then streaked onto LB plates supplemented with 850 mmol/L NaCl and adjusted to a pH of 8.5. All plates were then incubated at 30 °C for 24 h. The single colonies displaying a distinct morphology were selected and purified by repeated streaking. After further growth, these colonies’ individual purification was done on fresh LB solid medium. Their bacterial genomic DNA was extracted with a bacterial genomic DNA extraction kit (Shanghai Generay Biotech Co., Ltd., Shanghai, China). The amplified sequences generated by the universal 16S rRNA primer pair(515F/8060R) were sequenced and used to construct a phylogenetic tree via the neighbor-joining algorithm in MEGA 11 software [48]. Subsequently, the 16S rRNA sequences were aligned with type strains and representative strains from neighboring species were identified to obtain preliminary taxonomic tree information. This was followed by whole-genome phylogenetic reconstruction and verification through average nucleotide identity (ANI) (https://jspecies.ribohost.com/jspeciesws/) and DNA-DNA hybridization (dDDH) (https://ggdc.dsmz.de/ggdc.php#) to determine the precise taxonomic classification of strain G3.

### 4.2. Determination of P. fluorescens G3 Tolerance to Salt Stress

Colony properties of *P. fluorescens* G3 were observed on LB agar after incubation for 48 h at 30 °C. The salt tolerance ability of *P. fluorescens* G3 was determined on LB medium with NaCl concentrations that varied from 170 to 1360 mM. To gauge the suitable pH range for *P. fluorescens* G3’s growth, LB broth was used with an adjusted pH level of 6–10. The growth of *P. fluorescens* G3 was recorded at 600 nm after incubation at 30 °C for 6–72 h.

### 4.3. Assessing the PGP Traits of P. fluorescens G3

The strain’s ACC deaminase (ACCD) activity was quantified using a commercial ELISA detection kit (Shanghai Yuanju Biotechnology Co., Ltd., Shanghai, China). This assay detects the degradation of ACC by measuring the development of a yellow color, whose intensity correlates with the substrate concentration. The detection principle involved is based on a double-antibody sandwich ELISA. Briefly, micro-wells pre-coated with anti-ACCD antibodies were incubated sequentially with samples, standards, and horseradish peroxidase (HRP)-conjugated detection antibodies, with thorough washing steps implemented between incubations. Following the substrate (TMB) addition, a blue color develops but later turns yellow upon acidification. Absorbance (OD) was measured at 450 nm. All procedures were strictly followed according to the manufacturer’s instructions. Each treatment was set up with three biological replicates, and the data are presented as mean ± SEM

Phosphorus solubilization capacity was assessed using the method of Wan et al. [49]. Briefly, the purified *P. fluorescens* G3 was inoculated onto a MeHKNHa solid medium supplemented with either organic phosphorus or inorganic phosphorus sources. After incubation at 28 °C for 5 days, their respective solubilization capacity was quantified as the ratio of the halo zone diameter (D) to the colony diameter (d). Each treatment was set up with three biological replicates, and the data are presented as mean ± SEM.

Before conducting formal IAA quantification analysis, we preliminarily determined the IAA production by *P. fluorescens* G3 under tryptophan concentrations of 0, 1, 2, 3 and 4 g/L using the Salkowski colorimetric method. The results demonstrated that IAA production reached its peak level at the tryptophan concentration of 2 g/L. Therefore, to achieve optimal synthesis efficiency, this concentration was adopted for subsequent precise high-performance liquid chromatography (HPLC) measurements. Indole-3-acetic acid (IAA) production by *P. fluorescens* G3 was quantified using high-performance liquid chromatography (HPLC; Essentia LC-16, Shimadzu Co., Ltd., Shanghai, China), by following the method of Perrig et al. [50]. Briefly, bacterial cultures were grown on LB medium supplemented with 2 g/L of L-tryptophan (Macklin, Shanghai, China) for 2 days, at 30 °C and 180 rpm. This was followed by centrifugation at 12 000 g for 20 min at 4 °C. The resulting supernatant was adjusted to pH 2.5 with 1M HCl, extracted twice with two volumes of ethyl acetate, and the organic phase was vacuum-dried at 37 °C and then dissolved in methanol. The obtained extract could be used after filtration through a 0.22-μm membrane. All extracted samples were separated along a C18 analytical column (5 μm, 250 mm × 4.6 mm, Shimadzu) at 30 °C, using an injection volume of 10 μL. The mobile phase consisted of methanol–0.1% acetic acid (40/60, *v*/*v*); the analysis was performed at a flow rate of 1.0 mL/min for 40 min, with detection at 220 nm. Quantification was made using an external calibration curve generated from 3-Indoleacetic acid (standard) (HPLC > 98%; Shanghai Yuanye Bio-Technology Co., Ltd., Shanghai, China), ranging from 0 to 100 mg/L (R^2^ > 0.999). Each treatment was set up with three biological replicates, and the data are presented as mean ± SEM.

To determine extracellular polysaccharide (EPS) production, the total carbohydrate content was quantified using the sulfuric acid–phenol colorimetric method described by Dubois et al. Briefly, *P. fluorescens* G3 cultures were grown on LB medium for 2 days at 30 °C (with shaking at 180 rpm). After centrifugation at 5000 g for 10 min, 2 mL of supernatant was collected. To the supernatant, 1 mL of 6% phenol (*v*/*v*) was added, followed immediately by the addition of 2.5 mL of concentrated sulfuric acid. This mixture was thoroughly vortexed, then allowed to stand for 10 min, vortexed again, and finally incubated for a further 20 min at room temperature. Absorbance was measured at 490 nm with a spectrophotometer. A calibration curve was generated using glucose standards ranging from 0 to 1.2 μg/mL [51]. Each treatment was set up with three biological replicates, and the data are presented as mean ± SEM.

To analyze siderophore production, we used the Modified CAS Agar Medium Kit (Hopebio, Qingdao, China, CAS: chrome azurol sulphonate 10.87 g, 1 L distilled water) as per Schwyn et al. [52]. First, the *P. fluorescens* G3 strain was inoculated onto LB plates and incubated at 30 °C for 24 h. Then it was inoculated onto CAS medium and incubated at 30 °C for 3 days. An orange halo around colonies on blue agar plates indicates siderophore secretion. Each treatment was set up with three biological replicates, and the data are presented as mean ± SEM.

### 4.4. Effect of P. fluorescens G3 on Maize Growth Under Salt Stress

#### 4.4.1. Germination of Maize Seeds Under Salt Stress Conditions

Maize seeds were first rinsed 3 to 5 times with tap water, then sterilized with 75% ethanol for 2 min, followed by 2% NaClO for 15 min, and once more with 75% ethanol for 2 min, and finally rinsed again with sterile distilled water (3–5 times). Each sterilized seed was immersed in sterile water and the bacterial suspension for 2 h, and then allowed to dry naturally. After culturing *P. fluorescens* G3 on LB medium at 30 °C and 180 rpm for 24 h, its cells were centrifuged and collected; these were re-suspended in sterile water, and a final colony density of 108 CFU/mL was obtained. Seeds soaked in this bacterial suspension, or sterile water, five were placed in a Petri dish supplemented with 200 mmol/L NaCl. All dishes were transferred into a growth chamber (25 °C, 16/8 h light/dark cycle) for constant-temperature cultivation lasting 4 days. Seed germination was then observed, and the germination rate was calculated. The experiment was conducted using 30 maize seeds per treatment group. Each treatment was set up with three biological replicates, and the data are presented as mean ± SEM.

#### 4.4.2. Growth of Maize Seedlings Under Salt Stress Conditions

To investigate the effects of *P. fluorescens* G3 on maize seedlings, soil was also collected from the same site area (Lanzhou New District). We evaluated plants’ growth status by measuring their height, root length, stem diameter, fresh weight, and dry weight. The *P. fluorescens* G3 strain was cultured at 30 °C and 180 rpm for 24 h, then centrifuged to harvest its bacterial cells. These cells were resuspended in sterile water to prepare a bacterial suspension whose concentration was 5 × 10^8^ CFU/mL. Three days after transplanting the already germinated maize seeds (under the aforementioned conditions), 50 mL of the bacterial suspension was applied to each plant via root irrigation. That bacterial suspension was applied every 7 days, with the control (CK) group irrigated instead with 50 mL of water. Starting on the 7th day, each pot was irrigated with 50 mL of a solution that contained 200 mM NaCl to initiate salt stress, and this was applied every 2 days. Seedlings were harvested after 3 weeks of stress treatment for further analysis. We set up a separate CK group lacking inoculation with *P. fluorescens* G3 and salt stress. The pH values of soils from different treatment groups were measured with a pH meter using a soil-to-water ratio of 1:5 (*w*/*v*). Each treatment was set up with three biological replicates, and the data are presented as mean ± SEM.

#### 4.4.3. Biochemical Analysis of Maize Seedlings

The harvested seedlings that underwent 3 weeks of salt stress treatment were analyzed for six biochemical variables. Chlorophyll was extracted from leaves via the ethanol method to determine the chlorophyll content, while their proline content was measured using the proline (PRO) assay kit (Jiangsu Edison Biotechnology Co., Ltd., Nanjing, China). To estimate the enzyme activity of malondialdehyde (MDA), fungal catalase (CAT), superoxide dismutase (SOD), and peroxidase (POD), we used their corresponding assay kits (all from the Jiangsu Edison Biotechnology Co., Ltd.). Three replicates (individual potted plants) were used for each variable’s determination, with each measured three times. Each treatment was set up with three biological replicates, and the data are presented as mean ± SEM.

#### 4.4.4. Quantitative Analysis of Na^+^ and K^+^ Ions in Maize Seedlings

To evaluate the regulatory effects of *P. fluorescens* G3 upon ion homeostasis in maize seedlings under salt stress, their leaf and root samples were collected from the three different treatment groups: *P. fluorescens* G3-inoculated + 200 mM NaCl salt stress; non-inoculated + 200 mM NaCl salt stress; and lacking inoculation with *P. fluorescens* G3 and salt stress. Next, the harvested maize seedlings’ leaf and root samples were fully rinsed with deionized water to remove adhering soil particles and surface salts. Samples were oven-dried at 105 °C for 30 min to deactivate enzymes, and dried further at 70 °C until constant weight was achieved. These dried samples were thoroughly ground into fine powder using a mortar. From every leaf or root powder sample, we accurately weighed >1.0 g, placing each in a porcelain crucible for high-temperature ashing in an electric muffle furnace (Steps: maintain an initial temperature of 200 °C for 30 min; then increase the temperature at a rate of 5 °C/min to 550 °C; hold at this temperature for 4–6 h until the sample has completely ashed, turning a white or off-white color). Once the crucible cooled to room temperature, 5 mL of 1 mol/L nitric acid was added to it. We then let the mixture stand at room temperature for 1 h, or gently shook it to assist dissolution, to ensure the cations in the ash were fully dissolved in the acid solution. After shaking it well, we filtered each solution through a 0.22-μm microporous membrane to obtain a clear sample solution for formal testing. These were transferred into sterilized 10 mL centrifuge tubes for analysis by inductively coupled plasma mass spectrometry (ICP-MS) [53]. Each treatment was set up with three biological replicates, and the data are presented as mean ± SEM.

### 4.5. Whole-Genome Sequencing and Analysis of P. fluorescens G3

The genome of *P. fluorescens* G3 was sequenced on the BMK Cloud platform, with sequencing services implemented according to the standard PacBio protocol (including sample quality inspection, library construction, library quality assessment, and library sequencing). The applied sequencing principle is based on PacBio sequencing technology, which uses SMRT chips as the sequencing carrier. Inside the nanopores of the SMRT chip, DNA polymerase binds to the template, and four-color fluorescent labels are employed to mark the four types of bases (dNTPs). During the base-pairing phase, the incorporation of different bases emits distinct light signals; hence, the type of incorporated base can be reliably determined based on the measured wavelength and peak value of emitted light.

### 4.6. Quantitative Real-Time PCR (RT-qPCR) Analysis

The real-time quantitative polymerase chain reaction (RT-qPCR) technique was used in this study to precisely quantify the upregulated expression levels of candidate bacterial genes. For this, the 16S rRNA gene served as the internal reference, and the gene-specific primers’ information can be found in Table A1. The Total RNA Extraction Kit (Tiangen Biotech (Beijing) Co., Ltd., Beijing, China) was used to extract RNA from the cells, and this was then reverse-transcribed to cDNA, using the Prime Script RT Kit with DNA Eraser (Tiangen Biotech (Beijing) Co., Ltd.). To perform the RT-qPCR analysis, the SYBR Green dye method was utilized along with the ChamQ Universal SYBR qPCR Master Mix (Q711-02, Vazyme Biotech., Nanjing, China), in a real-time fluorescence quantitative PCR (qPCR) instrument (MA-6000, Molarray, China, Suzhou, China). Each RT-qPCR was conducted under these conditions: heat to 95 °C for 3 s; followed by 40 cycles at 95 °C for 10 s and 60 °C for 30 s; then maintain at 95 °C for 15 s, 60 °C for 60 s, and 95 °C again for 15 s. To quantify the relative amounts of mRNA, we relied on the 2^−∆∆Ct^ method in the calculation of gene expression levels. These experiments were conducted in triplicate, with resulting data presented here as the mean ± SE; their statistical analysis was carried out in GraphPad Prism 10.6.0 (San Diego, CA, USA).

### 4.7. Metagenomic DNA Extraction, Illumina Sequencing, and Assembly Analysis

The rhizosphere soil samples were collected 21 days after treating them with the bacterial solution. To do that, the maize seedlings were first removed from the pots, and then any soil loosely attached to their roots was gently shaken off. Soil within 1–2 mm of the root surface (i.e., rhizosphere soil) was collected into sterile cryotubes and stored at −80 °C. Each treatment group had three replicate soil samples (i.e., from three independent maize-grown pots). To extract their soil DNA, we used the Fast DNATM SPIN Kit for Soil (MP Biomedicals, Santa Ana, CA, USA) as per the manufacturer’s protocol. To avoid contamination from external substances during experiments, all procedures were performed in a sterile laminar flow hood. The DNA that passed the quality inspection was cryopreserved and sent to Beijing Biomarker Technologies Co., Ltd., for further sequencing of the soil bacterial community.

The raw reads (raw tags) obtained from Illumina sequencing were filtered by fastp to remove any low-quality sequences, yielding only high-quality clean reads (clean tags). Next, Bowtie2 was employed to align these clean reads against the maize reference genome while eliminating any host contamination. The ensuing clean reads were assembled de novo, using MEGAHIT, with those contigs shorter than 300 bp discarded to safeguard the assembly quality. To evaluate the assembly results, we used QUAST, which generated various key metrics, including contig number, total contig length, longest contig length, N50, and GC content. The coding DNA sequences (CDSs) were identified from the assembled contigs, using MetaGeneMark v3.26 with default parameters, to provide foundational data for their subsequent functional and taxonomic analyses. To minimize redundancies, predicted genes were clustered by MMseqs2 v12-113e3 at a protein sequence similarity threshold of 90% and a coverage threshold of 80%; hence, a non-redundant gene set was derived for downstream functional annotations (e.g., KEGG, COG) and taxonomic classifications.

### 4.8. Metagenomic Functional Annotation, Community Diversity Analysis

The obtained non-redundant gene set was annotated using multiple databases. General databases comprised Nr, GO, KEGG, eggNOG, Pfam, and SwissProt; specialized databases consisted of CARD, CAZy, and PHI-base. For the KEGG and eggNOG annotations, BLAST alignment was used (with an e-value threshold of 1 × 10^−5^); for the CAZy database annotation, enzyme families were identified by HMMER using hidden Markov models. We calculated and compared four alpha diversity indices-Chao1, Shannon, and Simpson-across the treatment groups to assess taxonomic richness and evenness. Principal component analysis (PCA) was carried out to explore similarities and differences in community structure between samples. We applied the LEfSe analysis (linear discriminant analysis effect size) with an LDA Score > 4.0, to screen for functionally significant differential genes (i.e., biomarkers) between groups. To run the RDA (redundancy analysis), we used the ‘vegan’ package (for R) to examine the relationships (correlations) between changes in functional gene abundance and environmental factors (i.e., plant physiological and biochemical indicators). To assess the intergroup differences in microbial taxa (presence/absence), we used the binary Jaccard distance, with this analysis conducted on the BMKCloud platform (https://international.biocloud.net/) (samples were compared across the three treatment groups: A–C, n = 3).

### 4.9. Statistical Analysis

Data were statistically analyzed in SPSS Statistics 27.0 software (IBM Corp., Armonk, NY, USA) and presented here as the mean ± SEM. To detect the differences in a given response variable among the treatment groups, one-way analysis of variance (ANOVA) was used, followed by Tukey’s HSD (honest significant difference) test for the pairwise comparison of means (at a significance level of *p* < 0.05). Among them, seed germination is a binary trait (germinated/non-germinated), and the difference in germination rate across treatment groups was analyzed using the Pearson chi-square test of independence for a contingency table. All expected cell frequencies in the contingency table were greater than 5, which meets the application assumptions of the chi-square test. Post hoc pairwise comparisons between treatments were performed on 2 × 2 sub-contingency tables, with Bonferroni correction applied to the significance level to account for multiple testing, and the significance threshold was set at *p* < 0.05.

## 5. Conclusions

This empirical study isolated the bacterial strain *P. fluorescens* G3, which is capable of effectively alleviating salt stress in maize through its salt-tolerance mechanisms (ion homeostasis and compatible solute synthesis), its plant growth-promoting (PGP) traits (phosphate solubilization, IAA production, ACC deaminase activity, EPS, and siderophore production), and its modulation of the rhizosphere microbial community. As a native strain, it demonstrates very promising application potential as a bio-agent to support maize production in saline–alkali soil regions of China’s Gansu Province, and perhaps elsewhere too, for promoting sustainable agriculture on these marginal lands.

## Figures and Tables

**Figure A5:**
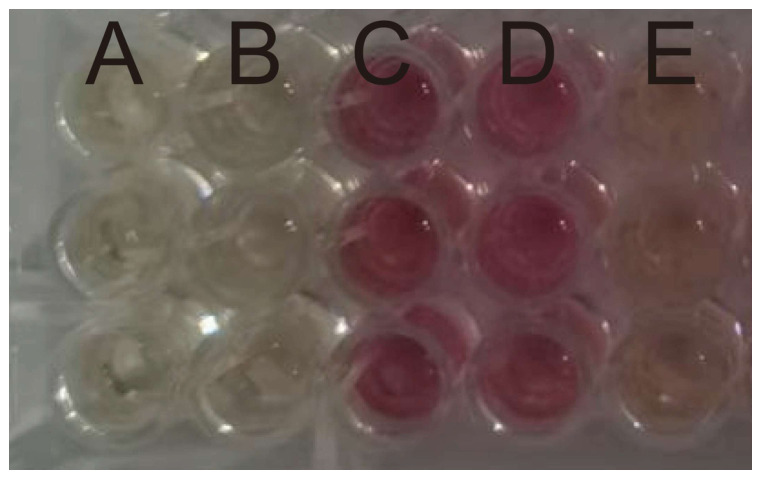
Before conducting formal IAA quantification analysis, we preliminarily determined the IAA production by *P. fluorescens* G3 under tryptophan concentrations of 0, 1, 2, 3 and 4 g/L using the Salkowski colorimetric method. Note: A: CK; B: 1 g/L L-tryptophan; C: 2g/L L-tryptophan; D: 3g/L L-tryptophan; E: 4g/L L-tryptophan.

## Data Availability

The metagenomic and strain whole genome data have been uploaded to NCBI with the following accession numbers: BioProject ID [PRJNA1402034], sequence accession number [JBUXES010000000].

## Appendix A

**Table A1:** Salt-tolerant genes related to strain G3’s whole genome and their gene descriptions.

Trait	Gene	Gene Function
Synthesis Transport	*proV*	glycine betaine/proline transport system ATP-binding protein
	*proW*	glycine betaine/proline transport system permease protein
	*proP*	MFS transporter, MHS family, proline/betaine transporter
Ion Homeostasis Stress Metabolism	*trkA*	trk system potassium uptake protein
	*nhaA*	Na^+^:H^+^ antiporter, NhaA family
	*sodA*	superoxide dismutase, Fe-Mn family
	*sodB*	superoxide dismutase, Fe-Mn family
Signal Transduction Regulation	*envZ*	two-component system, OmpR family, osmolarity sensor histidine kinase EnvZ
	*ompR*	two-component system, OmpR family, phosphate regulon response regulator OmpR

**Table A2:** Primer pairs of candidate genes used for the validation by RT-qPCR.

Gene	Forward Primer Sequences (5′-3′)	Reverse Primer Sequences (5′-3′)
*16S rRNA*	gcgaccacctggactgata	aaggctcccaacggcta
*proV*	ccagtggcgcaacaaga	aacggttcgtccatcagc
*proW*	tgatgccgttgctggac	gatcaccgtggcgaaaat
*proP*	gctgggacgcaagaaaat	tcacccgcatcaacaaca
*trkA*	gccgtgatgagcgaaatg	ctcgatgatcttgacctggtag
*nhaA*	acgaacaggctcatggtga	tggctggccgtgaaaat
*sodA*	gcggtatcgagcgtttca	ttccaccatcagggtctttt
*sodB*	ataccgcccgaggaagtct	ggaataccaccacgacaagc
*envZ*	ttgaaacgcctggtctacg	cagcctgttccacatcctc
*ompR*	tactgcgtcgccaatcg	gagcatgtggacctcatcg

**Table A3:** DEGs related to G3’s alleviation of salt stress.

Gene ID	Gene Name	Regulate	Log2FC
GE005195	*proV*	up	2.5
GE000416	*proW*	up	2.8
GE000126	*proP*	up	2.3
GE002421	*trkA*	up	2.6
GE003959	*nhaA*	up	2.9
GE000872	*sodA*	up	2.2
GE001277	*sodB*	up	2.7
GE000309	*envZ*	up	2.4
GE003550	*ompR*	up	2.6
